# *ONECUT1* variants beyond type 1 and type 2 diabetes: exploring clinical diversity and epigenetic associations in Arab cohorts

**DOI:** 10.3389/fgene.2023.1254833

**Published:** 2023-10-24

**Authors:** Mohammed Dashti, Rasheeba Nizam, Sumi Elsa John, Motasem Melhem, Arshad Channanath, Hessa Alkandari, Thangavel Alphonse Thanaraj, Fahd Al-Mulla

**Affiliations:** ^1^ Department of Genetics and Bioinformatics, Dasman Diabetes Institute, Kuwait City, Kuwait; ^2^ Department of Specialized Services Facility, Dasman Diabetes Institute, Kuwait City, Kuwait; ^3^ Department of Population Health, Dasman Diabetes Institute, Kuwait City, Kuwait; ^4^ Department of Pediatrics, Farwaniya Hospital, Ministry of Health, Kuwait City, Kuwait

**Keywords:** *ONECUT1*, SNP, MODY, T1D, T2D, Arab ethnicity, differential methylation profiles, different types of diabetes

## Abstract

*ONECUT1* gene, encoding hepatocyte nuclear factor 6, is involved in pancreas and liver development. *ONECUT1* mutations impair the function of pancreatic β-cells and control a transcriptional/epigenetic machinery regulating endocrine development. Homozygous nonsense and missense mutations at *ONECUT1*_p.E231 and a homozygous frameshift mutation at *ONECUT1*_p.M289 were reported in neonatal diabetes individuals of French, Turkish, and Indian ethnicity, respectively. Additionally, heterozygous variants were observed in Northern European T2D patients, and Italian patients with neonatal diabetes and early-/late-onset T2D. Examining diverse populations, such as Arabs known for consanguinity, can generalize the *ONECUT1* involvement in diabetes. Upon screening the cohorts of Kuwaiti T1D and MODY families, and of Kuwaiti and Qatari T2D individuals, we observed two homozygous variants—the deleterious missense rs202151356_p.H33Q in one MODY, one T1D, and two T2D individuals, and the synonymous rs61735385_p.P94P in two T2D individuals. Heterozygous variants were also observed. Examination of GTEx, NephQTL, mQTLdb and HaploReg highlighted the rs61735385_p.P94P variant as eQTL influencing the tissue-specific expression of *ONECUT1*, as mQTL influencing methylation at CpG sites in and around *ONECUT1* with the nearest site at 677-bases 3′ to rs61735385_p.P94P; as overlapping predicted binding sites for NF-kappaB and EBF on *ONECUT1*. DNA methylation profiles of peripheral blood from 19 MODY-X patients versus eight healthy individuals revealed significant hypomethylation at two CpG sites—one located 617-bases 3′ to the p.P94P variant and 8,102 bases away from transcription start; and the other located 14,999 bases away from transcription start. Our study generalizes the association of *ONECUT1* with clinical diversity in diabetes.

## Introduction

One Cut Homeobox 1 (*ONECUT1*), also known as hepatocyte nuclear factor 6 (HNF6), is a pancreatic transcription factor that influences various cellular processes, including glucose metabolism. It belongs to a family of genes that encode key proteins involved in pancreas and liver development. ONECUT1 plays a crucial role in regulating the fate of pancreatic progenitor cells (Heller et al., 2021). Recent research suggests that genetic variations in the ONECUT1 gene contribute to various forms of diabetes by impairing the function of insulin-producing pancreatic *β*-cells; Philippi et al. (2021) presented an exemplary study on this subject, wherein they observed rare variants within the *ONECUT1* gene in individuals with monogenic diabetes (recessive and dominant) and multifactorial type 2 diabetes (T2D). The authors observed the following *ONECUT1* variants: i) a homozygous protein-truncating variant, p.E231X, found in a French boy (from a consanguineous family) with severe neonatal syndromic diabetes; ii) a homozygous missense variant, p.E231D, found in a Turkish boy (from a consanguineous family) diagnosed with insulin-requiring diabetes at the age of 14 months; and iii) a set of six heterozygous missense variants in 13 T2D patients, with an early disease onset than noncarriers, of predominantly Northern European ancestry from the Ulm Diabetes Cohort (UDC-T2D cohort); these six variants included p.H33Q, p.G30S, p.G62C, p.P75A, and p.V242A showing association with increased risk of T2D, and p.G96D showing a protective effect but having a modifier effect from p.P75A. A study by Prudente et al. (2022), subsequent to that of Philippi et al. (2021), investigated the role of *ONECUT1* variants in Italian patients with various forms of nonautoimmune diabetes mellitus, including permanent neonatal diabetes mellitus (PNDM), familial diabetes, and early- and late-onset T2D. The researchers identified 13 *ONECUT1* heterozygous variants in patients with different subtypes of diabetes. A more recent report by Russ-Silsby et al. (2023), identified a homozygous frameshift variant in ONECUT1 as the cause of neonatal diabetes in one individual referred from India to Exeter Monogenic Diabetes Cohort; they further found, by way using a rare variant burden test in the UK Biobank European cohort, a significant association between heterozygous ONECUT1 null variants and type 2 diabetes. The findings from the above-mentioned three studies confirm the critical role of ONECUT1 in human beta-cell function.

Further to examining genetic variants from *ONECUT1*, Philippi et al. (2021) dissected the functional consequences of defective protein encoded by *ONECUT1* in pancreatic development, by way of using genome-edited human embryonic stem cells and patient-specific induced pluripotent stem cells. Their study revealed that loss of *ONECUT1* altered transcription factor binding and enhancer activity and NKX2.2/NKX6.1 expression in pancreatic progenitor cells resulting in impaired pancreatic progenitor formation and an impaired subsequent endocrine program. Collectively, they demonstrated that *ONECUT1* controls a transcriptional and epigenetic machinery regulating endocrine development, involved in a spectrum of diabetes, Studies involving additional populations can help to further confirm the contribution of *ONECUT1* variants to different diabetes types. The Arab population is known for the common practice of consanguineous marriages and inbreeding (Tadmouri et al., 2009; Al-Gazali and Hamamy, 2014). Arab countries, such as Kuwait, have high prevalence of diabetes and metabolic disorders (Channanath et al., 2013; Shaltout et al., 2017; Abu-Farha et al., 2021; Al-Kandari et al., 2021; Elashi et al., 2022). Consanguineous marriages among Arabs often involve cousins or relatives within large extended families or the same tribe, with consanguinity rates reaching as high as 54.3% (Al-Awadi et al., 1985). This practice of consanguinity has led to increased endogamy, homozygosity, and accumulation of deleterious recessive alleles in the gene pool, making the population susceptible to various recessive genetic disorders. Consanguinity and inbreeding may also play a major role in the aetiology of complex disorders, such as diabetes (Rudan et al., 2006), with autosomal recessive alleles potentially playing a larger role in the pathogenesis of complex traits in this population (Bittles and Black, 2010). These deliberations on the population history of Arabs motivated us to explore the broader contribution of *ONECUT1* in this indigenous population. In this study, we examined the coding variants from *ONECUT1*, by way of screening our in-house exome datasets comprising diabetic individuals of Arab ethnicity. These datasets included i) exome data from Kuwaiti families with one or more members diagnosed for maturity-onset diabetes of the young (MODY), ii) exome data from Kuwaiti families with one or more members diagnosed for type 1 diabetes (T1D), and iii) exome data from individuals diagnosed for T2D. We also examined publicly available exome datasets of T2D individuals from Qatar. Publicly available HaploReg databases were used to examine whether the *ONECUT1* variants are part of transcription factor binding sites. The GTEx and NephQTL databases were examined to assess whether the *ONECUT1* variants have genetic influence on tissue-specific gene expression. Further, to examine the epigenetic involvement of the *ONECUT1* variants in diabetes, we used mQTLdb to examine whether the observed *ONECUT1* variants are QTLs for methylation sites in and around the gene region. Additionally, we examined the DNA differential methylation profiles of peripheral blood from MODY patients.

## Methods

### Ethics statement

The study protocol was approved by the Ethical Review Committee of Dasman Diabetes Institute in accordance with the guidelines of the Declaration of Helsinki and the United States Federal Policy for the Protection of Human Subjects. The cohorts corresponding to the exome datasets examined in this study were recruited as part of earlier published studies, and no participant recruitment took place as part of the current study. At the time of recruitment, written informed consent was obtained from the individuals; in the case of minor individuals, consent was obtained from the parent(s) and assent was obtained from the minor individuals.

### Datasets

We analyzed the *ONECUT1* variants in individuals of Arab ethnicity, by way of using our in-house exome datasets of diabetes patients from Kuwait and publicly available exome datasets of diabetes individuals from Qatar. The datasets consisted i) exomes of 47 MODY patients and 32 healthy individuals from 32 Kuwaiti families with one or more members diagnosed with MODY (Al-Kandari et al., 2021); ii) exomes of 63 T1D patients and 62 healthy individuals from 35 Kuwaiti families with one or more members diagnosed with T1D; iii) exomes of 122 T2D patients and 169 healthy individuals from a T2D cohort in Kuwait (John et al., 2018); and iv) publicly available exomes from 503 T2D patients and 261 healthy individuals from Qatar (O’Beirne et al., 2018). Additionally, we performed targeted sequencing of *ONECUT1* on an additional sample set of 88 T2D individuals from Kuwait. In order to confirm the genotype calls at examined variants from *ONECUT1* in the exome datasets, we performed targeted genotyping on samples displaying genotypes that are heterozygous or homozygous for minor allele at selected variants.

### Use of publicly available annotation databases

Transcription factor binding motifs, from Haploreg (Ward and Kellis, 2012), overlapping the position of the *ONECUT1* variants were identified using the T1D Knowledge Portal (Kudtarkar et al., 2023). Position weight matrices used in the tool are as derived by Kheradpour and Kellis (Kheradpour and Kellis, 2014) using regulatory motifs in ENCODE transcription factor binding experiments. Possible roles of variants in disease aetiology were investigated by analysing data on tissue-specific expression of the quantitative trait locus (eQTL) from resources such as the NEPTUNE patient characteristics for subjects in expression quantitative trait loci (NephQTL) (Gillies et al., 2018) and genotype-tissue expression (GTEx) (GTEx Consortium, 2017) databases. The mQTLdb (Gaunt et al., 2016) (http://www.mqtldb.org/), which is a public resource on lead mQTL loci influencing methylation variation, in children at birth, childhood, adolescence and their mothers during pregnancy and middle age, was used to examine the genetic influence of the *ONECUT1* variants on differential methylation at CpG sites at different life stages.

### Differential methylation analysis

The methodologies for methylation sequencing and DNAm analysis are described in detail in our previous publication (Dashti et al., 2022). A brief summary of the adopted methods is as given below**.** Methylome libraries were prepared using TruSeq Methyl Capture EPIC Library Prep Kit reagents (Illumina Inc., United States) using the protocol provided by the manufacturer. Indexed libraries were clustered on a cBOT system using TruSeq paired cluster kit V3 (Illumina Inc., United States), and sequencing of paired-end reads was performed on an Illumina HiSeq 2500 sequencing platform. High-quality paired-end trimmed reads, generated using Trim Galore! Version 0.3.3 (Babraham Bioinformatics, United Kingdom), were mapped to the *in silico* UCSC human assembly HG19 reference genome using Bismark (version 0.22.3) (Krueger and Andrews, 2011) and Bowtie2 (version 2.3.5.1) (Langmead and Salzberg, 2012). Single CpG site analysis was performed on the resulting sequence alignment/map (SAM) files using the methylKit package version 1.2.4 (Akalin et al., 2012). Differentially methylated single CpG sites were detected if the methylKit *q-value* is ≤0.01 and the difference in the methylation levels between the compared groups was ideally ≥25%; and additional sites exhibiting less differences in methylation levels were considered, as long as the *q-value* criteria is satisfied.

## Results

### Homozygous and heterozygous *ONECUT1* variants observed in diabetes individuals

**FIGURE 1:**
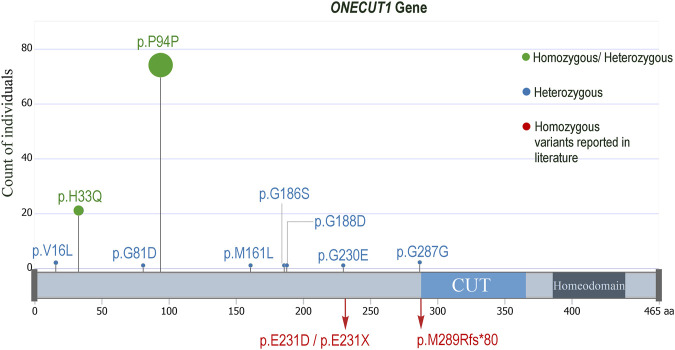
Lollypop mutation plot displaying the *ONECUT1* variants seen in the examined diabetes datasets from Kuwait and Qatar. The p.H33Q and the p.P94P were seen in homozygous as well as in heterozygous forms, while all the others were seen only in heterozygous forms. The homozygous mutations observed by Philippi et al. (2021), Russ-Silsby et al. (2023) are also indicated.

Upon examining the study exome datasets of individuals with MODY, T1D and T2D for variants from *ONECUT1*, we observed two homozygous variants, namely, the deleterious missense rs202151356_ p.H33Q, and the synonymous rs61735385_p.P94P (Table 1). The first one was observed in one Kuwaiti individual with MODY, one Kuwaiti individual with T1D, and in two Qatari individuals with T2D. The second homozygous variant was observed in two Qatari individuals with T2D. The minor allele frequency of the rs202151356_ p.H33Q in the five continental populations are particularly very low with a value of ≤0.3% while that of rs61735385_p.P94P is >5% in Europeans and South Asians, and <1% in East Asians. These two variants were also observed in heterozygous form in additional patients from Kuwait and Qatar (see Table 1). Further, we observed two heterozygous variants, namely, rs866368632_5′ UTR (in one Kuwaiti individual with MODY) and rs201286990_p.M161L (in one Kuwaiti individual with T2D) (see Table 1; Figure 1). Both these two heterozygous variants were not seen in continental populations but seen in Middle East (rs866368632_5′ UTR with an MAF of 0.3%) or in Ashkenazi Jews (rs201286990_p.M161L with an MAF of 0.7%). In addition to the variants mentioned above, Qatari T2D patients exhibited the following heterozygous variants: rs2075613_p.G287G (2 individuals), rs147745937_p.G230E (1 individual), rs151292910_p.G188D (1 individual), rs1483480013_p.G186S (1 individual), rs142641519_p.G81D (1 individual), and rs760541486_p.V16L (2 individuals). None of these variants was observed in the datasets of the Kuwaiti cohort, except that the rs2075613_p.G287G was seen in two healthy individuals from Kuwait.

**TABLE 1:** Variants identified in the coding region of *ONECUT1* gene in patients with different subtypes of diabetes mellitus. The corresponding transcript identifier is NM_004498.4.

SNP identifier of the observed variant^a^	Nucleotide change in the transcript (and on DNA)	Consequence	MAF (from 1000 genomes project phase 3 or from gnomAD exome)	Number of afflicted individuals with the variant observed in the different diabetes subtypes. Homo: genotype at the variant is homozygous for the minor allele; hetero: genotype at the variant is heterozygous for the minor allele			SIFT classification
				MODY Kuwait (*n* = 47)	T1D Kuwait (*n* = 63)	T2D Kuwait (*n* = 122)/Qatar (*n* = 503)	
rs202151356	c.99C > A (G > T)	missense p.H33Q	AFR:0.003	homo:1	homo:1	homo:2 (Qatar)	Deleterious (Sift score 0.02)
			AMR:0.001				
			EAS:0.000	hetero:5	hetero:3	hetero:9 (Qatar)	
			EUR:0.002				
			SAS:0.000				
rs61735385	c.282G > C (G > C)	synonymous p.P94P	AFR:0.014	hetero:1	hetero:6	homo:2 (Qatar)	Benign
			AMR:0.027				
			EAS:0.005			hetero:65 (Qatar)	
			EUR:0.087				
			SAS:0.091				
rs866368632	c.-72C > T (G > A)	Promoter regulatory region variant 5′ UTR.	AFR:0.000	hetero:1			
			AMR:0.00006				
			EAS:0.000				
			NFE:0.000				
			SAS:0.000				
			MID:003				
rs201286990	c.481A > C (T > G)	missense p.M161L	AFR:0.000			(hetero: 1 (Kuwait)	Deleterious (Sift score 0.03)
			AMR:0.000				
			EAS:0.000				
			NFE:0.00004				
			SAS:0.000				
			ASJ:007				

^a^ In addition to the variants listed in Table 1, individuals with T2D in the Qatari cohort showed the following variants, all with a heterozygous genotype: rs2075613_p.G287G (2 individuals), rs147745937_p.G230E (1 individual), rs151292910_p.G188D (1 individual), rs1483480013_p.G186S (1 individual), rs142641519_p.G81D (1 individual), and rs760541486_p.V16L (2 individuals). None of these variants were observed in the datasets of the Kuwaiti cohort.

### Familial aggregation

Familial aggregation of diabetes in Kuwaiti families is amply demonstrated in the pedigrees considered in this study–for example, family history of the MODY patient exhibiting the rs202151356_p.H33Q variant indicates familial aggregation of diabetes in both the maternal and paternal first-degree relatives (Supplementary Figure S1A). The rs202151356_p.H33Q variant was observed (either as homozygous or heterozygous) in four patients with diabetes from three unrelated MODY families (Table 2). Two of these four patients with MODY were siblings. The grandparent of another patient with MODY had been diagnosed with T1D, while the entire family has a high prevalence of T2D. These four patients did not carry mutations and/or structural variants in known MODY genes, as confirmed by using whole-exome sequencing and Multiplex ligation-dependent probe amplification assay, respectively. Moreover, two of these four individuals tested negative for glutamic acid decarboxylase (GAD) and islet antigen 2 (IA2) autoantibodies, while the other two tested positive for GAD and IA2 autoantibodies. Furthermore, the individual who exhibited the homozygous TT genotype had an onset of diabetes at an age in the range of 0–3 years, while the three individuals with the heterozygous GT genotype had onset age in the range of 8–14 years.

**TABLE 2:** *ONECUT1*: rs202151356_p.H33Q) variant observed in datasets of Arab individuals with diabetes.

Participant number^a^	Nationality	Sex	Age at onset (range in years)^b^	Genotype at rs202151356 in the patient
MODY dataset^c^				
1	Kuwaiti	F	0–3	TT
2	Kuwaiti	M	12–15	GT
3 (sibling of 2)	Kuwaiti	F	12–15	GT
4	Kuwaiti	M	6–9	GT
T1D dataset^d^				
5	Kuwaiti	M	6–9	TT
6	Kuwaiti	F	0–3	GT
7 (sibling of 6)	Kuwaiti	M	3–6	GT
8 (sibling of 5)	Kuwaiti	M	Unaffected at the time of recruitment	GT
T2D dataset (Qatar)^e^				
SRR2130824	Qatari	F	NA	TT
SRR2130968	Qatari	F	NA	TT
SRR2125404	Qatari	M	NA	GT
SRR2130480	Qatari	M	NA	GT
SRR2130606	Qatari	F	NA	GT
SRR2130632	Qatari	F	NA	GT
SRR2130673	Qatari	F	NA	GT
SRR2130726	Qatari	F	NA	GT
SRR2130965	Qatari	F	NA	GT

^a^ The participant numbers listed for the individuals from MODY, and T1D datasets are not biobank sample identifiers; they are masked identifiers assigned for the purpose of referring within the manuscript text; these numbers do not represent identifiable information of the participant. The numbers listed for the individuals from the Qatari T2D dataset are not sample identifiers; they are published sequence accession numbers from the NCBI, sequence read archive.

^b^ It is not possible to give the exact age at onset as it is considered as indirectly identifiable patient/participant data.

^c^ The Kuwaiti MODY, cohort comprises 32 families with 47 cases and 32 controls who underwent exome sequencing. The variant was not observed in any of the controls from these families.

^d^ The Kuwaiti T1D cohort comprises 35 families with 63 cases and 62 controls who underwent exome sequencing. The variant was not observed in any of the 62 controls (except one with participant number 8) from these families. The age of participant number 8 at the time of recruitment was in the range of 8–10 years, while the age of their father at T1D onset was in the range of 25–28 years, that of their grandparent was in the range of 18–21 years, and sibling was in the range of 6–9 years. It is possible that the child developed T1D after the recruitment.

^e^ The Qatari T2D cohort (764 total number of participants comprising 503:T2D and 261:control) was as reported in the study by O’Beirne et al. (2018) and was downloaded from the National Center for Biotechnology Information Sequence Read Archive (SRA, accessions SRP060765, SRP061943, and SRP061463). The heterozygous GT, genotype for the variant was also observed in nine individuals from the Qatari cohort without T2D at the time of recruitment. The variant was not observed in the considered exome datasets of 122 individuals with T2D from Kuwait. additionally, targeted sequencing of 88 T2D individuals from Kuwait failed to detect the variant. However, in two of the 169 Kuwaiti individuals without diabetes, a GT, genotype was observed at the variant. One of these two individuals, with age in the range of 65–70 at the time of recruitment, had an FPG, level of 6.6 mmol/L, indicative of prediabetes, and had been suffering with hypertension for 20 years; the other individual was aged only 18–20 years at the time of recruitment.

In the T1D dataset, the rs202151356_p.H33Q variant was observed in three individuals with T1D from two unrelated T1D families in Kuwait. Among these three individuals with T1D, the homozygous TT genotype was observed in one person, with an age at onset in the range of 6–9 years; the other two individuals exhibiting heterozygous GT genotype were siblings with age at onset in the range of 0–3 years and 3–6 years. Additionally, a fourth individual (participant number 8 in Table 2), unaffected at the time of recruitment, also displayed the GT genotype at the variant (see Supplementary Figure S1B for the pedigree).

### Transcription factor (TF) binding motifs altered by the *ONECUT1* variants

Upon examining the transcription factor binding motifs from HaploReg (see *Methods*), it was found that both the homozygous variants overlapped the transcription factor binding motifs (known or predicted using motifs in ENCODE transcription factor binding experiments). The mutations at these sites altered the score (information content) of the motifs by up to >11 bits (Supplementary Table S1). Alternate allele at the homozygous rs202151356_p.H33Q variant reduced the score of the TF motifs Myc_disc9 and Myc_known8; alternate allele at the other homozygous rs61735385_p.P94P variant reduced the scores of TF motifs NF-kappaB_disc2 and EBF_known3. Of the two heterozygous variants seen in Kuwaiti diabetes individuals (see Table 1), the rs201286990_p.M161L overlapped with the TF motif of Mef2_known6 and the alternate allele reduced the score by up to 12 bits.

### Impact of the observed *ONECUT1* variants on differential expression levels of genes

Examination of GTEx to determine whether the observed variants exhibit genotype-based differential expression of genes revealed that the rs61735385_p.P94P significantly downregulates the expression levels of *ONECUT1* (*p-value* = 5.2E−07) and *RP11-209K10.2* (*p-value* = 1.1E−06) genes in testis (Supplementary Table S2). Examination of the NephQTL database to determine which of the lead variants act as eQTL in kidney revealed that the rs61735385_p.P94P exhibited genotype-based differential expression (downregulation) of MYO5A, MYO5C, ONECUT1, FAM214A, and ARPP19 genes in either glomerular or tubular tissues; however, the *p*-values for the t-statistics were not significant (Supplementary Table S2).

### Impact of the observed *ONECUT1* variants on differentially methylated CpG sites

Examination of the mQTLdb revealed that the rs61735385_p.P94P has genetic influence on DNA methylation at various CpG sites, in or around the *ONECUT1* gene region, during different life stages (Supplementary Table S3). The nearest CpG site, namely, cg02061705, is located 677 bases away within the gene from the rs61735385_p.P94P variant; and is differentially methylated in adolescent stage of the life cycle. The farthest CpG is located 19,748 bases downstream to the position of the variant.

### Examining DNA methylation profiles of peripheral blood between cases and controls from our familial MODY cohort

We examined data from our ongoing methylation study to determine whether differential methylation events occur in response to diabetes in the genome region harbouring the *ONECUT1* gene. We focused on patients with MODY-X non-autoimmune diabetes, who were clinically suspected as having MODY, as per the International Society for Pediatric and Adolescent Diabetes (ISPAD) criteria, and responded well to diabetes treatment, but did not exhibit variants of significance in any known monogenic diabetes-associated genes. By comparing DNA methylation (DNAm) profiling of peripheral blood from 19 individuals with MODY-X (including three patients with the rs202151356_p.H33Q variants) with DNAm profiling of eight control individuals, we found two significantly differentially methylated CpG sites at Chr15:53058083 (*q-value* = 3.75E−40), and Chr15: 53081183 (*q-value* = 0.008) (Table 3). Both the sites are hypomethylated in MODY cases, the first one with 35% difference in methylation and the second one with close to 10% difference. This second site, located at Chr15:53081183 is particularly interesting as it is mere 800 bases away from the rs202151356_p.H33Q variant (Chr15:53081983) and 617 bases away from the rs61735385_p.P94P variant (Chr15:53081800). The first site, located at Chr15:53058083 is located 23,900 bases and 24,083 bases away from the rs202151356_p.H33Q and the rs61735385_p.P94P variants, respectively. The first site is located 14,999 bases away from the transcription site and the second site is located at 8,102 bases away from the transcription site.

**TABLE 3:** CpG sites differentially methylated in *ONECUT1* gene between individuals with MODY-X and healthy individuals at a *q-*value ≤ 0.01 for differences in methylation levels.

CpG sites	*q*-value	Difference in methylation levels (in %)	Distance to the rs202151356_p.H33Q (15:53081983)	Distance to the rs61735385_p.P94P (15:53081800)	Distance to transcription site
Chr15:53058083	3.75E-40	−35.1973	23,900 bases	24,083 bases	14,999 bases
Chr15: 53081183	0.007887	−9.86426	800 bases	617 bases	8,102 bases

## Discussion

Our study observed two homozygous ONECUT1 variants in Arab patients of diabetes—the deleterious missense rs202151356_p.H33Q was observed in 1 individual of MODY, 1 individual of T1D and two individuals of T2D; and the synonymous rs61735385_p.P94P was observed in two T2D individuals. These variants were observed with heterozygous genotypes as well in additional patients. None of the individuals without diabetes (healthy controls) from the T1D or MODY families carried these variants, except for one individual (participant number 8, as mentioned in Table 2, carrying a GT heterozygous genotype at rs202151356_p.H33Q), and is a sibling of a T1D proband with homozygous genotype) who was not diagnosed with T1D at the time of recruitment (see Supplementary Figure S1B). The age at the time of recruitment of this unaffected participant was in the range of 8–10 years; considering that the age at onset of T1D in the grandparent was in the range of 18–21 years, in the father was in the range of 25–28 years, and in the sibling was in the range of 6–9 years, it is possible that the child developed T1D after recruitment.

Prudente et al. (2022) previously reported these two variants in heterozygous forms in Italian patients: the rs202151356_p.H33Q in individuals with early-onset T2D individuals; and the rs61735385_p.P94P in individuals with familial diabetes of adulthood, early-onset T2D, and late-onset T2D. Philippi et al. (2021) also observed the rs202151356_p.H33Q variant in heterozygous form in three T2D individuals of Northern European ancestry from the UDC-T2D cohort. For the first time, we report the presence of these two variants homozygous for the minor allele in individuals with MODY, T1D, or T2D. The rs202151356_p.H33Q is a very rare variant in all the five continental populations, while rs61735385_p.P94P is a common variant in both the European and South Asians while it is rare in East Asians. The SIFT tool predicted the rs202151356_p.H33Q to be deleterious.

Apart from the two homozygous variants, the Kuwaiti datasets revealed two heterozygous variants, namely, the rs866368632_5′UTR in a MODY individual, and the rs201286990_p.M161L in a T2D individual. The Qatari T2D dataset revealed the following 6 heterozygous variants, namely, the rs2075613_p.G287G rs147745937_p.G230E, rs151292910_p.G188D, rs1483480013_p.G186S, rs142641519_p.G81D, and rs760541486_p.V16L. Prudente et al. (2022) had observed two of these heterozygous variants in their study: the rs1483480013_p.G81D variant in an individual with permanent neonatal diabetes and the rs2075613_p.G287G in individuals with permanent neonatal diabetes, familial diabetes of adulthood, and late-onset T2D.

While the rs202151356_p.H33Q is a deleterious missense variant, the rs61735385_p.P94P seems to have implications on transcription-related aspects as indicated by the following findings of our study: i) Both the homozygous variants overlapped the predicted transcription factor (TF) binding motifs (known or predicted using motifs in ENCODE TF binding experiments) on *ONECUT1*. Alternate allele at the homozygous rs202151356_p.H33Q variant reduced the score of motifs for the binding of TF Myc; alternate allele at the other homozygous rs61735385_p.P94P variant reduced the scores of TF motifs for the binding of transcription factors NF-κB and EBF. It is known that Myc has a potential physiological role on the regulation of the β-cell function (Rosselot et al., 2021). NF-κB activation is a key event involved in diabetes pathogenesis (Patel and Santani, 2009). ii) The genotype-tissue specific expression databases indicated that the rs61735385_p.P94P significantly downregulates the tissue-specific expression levels of *ONECUT1* and *RP11-209K10.2* genes. The study of Philippi et al. (2021) demonstrated that one of the endocrine regulatory elements affected by T2D-associated variants near *ONECUT1* is the above-mentioned lncRNA RP11-209K10.2, and that the lncRNA exhibits similar tissue-specific expression as *ONECUT1*. iii) The rs61735385_p.P94P is a mQTL regulating the DNA methylation at CpG sites, in or around the *ONECUT1* gene region, during different stages of life. The nearest CpG site, is located a mere 677 bases away within the gene from the rs61735385_p P94P variant; and is differentially methylated in adolescent stage of the life cycle.

Further, our data from comparing the DNA methylation profiles of MODY-X nonautoimmune diabetes patients and healthy individuals suggested an epigenetic involvement of *ONECUT1* gene *via* differential DNA methylation in patients with MODY-X. The *ONECUT1* emerged as one of the top hypomethylated genes in MODY-X. Of the two observed statistically significant hypomethylation CpG sites in ONECUT1 in MODY-X, one was located 617 bases away from the rs61735385_p.P94P variant. The other CpG is located 14,999 bases away from the transcription start; and is located in an intron within the gene body. The *ONECUT* gene region is known to exhibit an altered DNA methylation pattern in response to stimuli. For instance, in mice that were fed a high-fat diet, Zhang et al. (2017) observed a significant suppression of *ONECUT1* expression in the RNAseq data and differential methylation of CpG sites at multiple sites within 10,000 bp of the transcription start region. Since ONECUT1 is a pancreatic transcription factor, differential methylation events may directly impact the cellular mechanisms that regulate the disease model (Thompson et al., 2015). The work by Heller et al. (2021) reported that *ONECUT1* plays a crucial role in pancreatic and endocrine development, and its transcriptional activity is intricately linked to its methylation status. The study by Rimoldi et al. (2022) underscored the interconnections between DNA methylation profiles and transcription factor binding. Our findings further support this association, particularly in the context of diabetes.

There is considerable familial aggregation of diabetes in Kuwaiti population (see Supplementary Figure S1A for an illustration). Our study illustrates familial aggregation of these risk variants: two of the four MODY probands with the homozygous or heterozygous rs202151356_p.H33Q variants are siblings, and two of the three T1D probands with the homozygous or heterozygous rs202151356_p.H33Q variants are siblings (see Table 2). Performing studies such as ours addressing aspects of molecular diagnosis for patients represent a very important task for families.

Over the past few decades, diabetes has evolved to a complex multifactorial disease, characterized by a wide range of clinical and genetic heterogeneity. The dichotomous classification of diabetes as T1D and T2D has been overruled by several distinct subtypes that differ in terms of clinical presentation, genetic variations, penetrance, and disease loci. While T1D and T2D are the most prevalent forms of diabetes in the Arabian Peninsula, the region also accounts for a vast number of MODY cases. Mutations in 14 different genes have been associated with the aetiology of MODY, defining its specific subtypes. Mutations in *GCK*, *HNF1A*, *HNF1B*, and *HNF4A* account for approximately 80% of the MODY cases (Juszczak et al., 2016). Despite this, a large proportion of suspected MODY cases, referred to as MODY-X, remain unsolved, with no variants of significance detected in known MODY genes. Interestingly, our study highlights *ONECUT1* methylation as a potential marker for MODY-X, representing either a unique subtype of MODY or a form of diabetes that is yet to be classified. In this context, it is important to point out that our previous study (Dashti et al., 2022) did not find *ONECUT1* as one of the observed differentially methylated and expressed genes in familial T1D. Thus, the observed *ONECUT1* methylation in the MODY patients in this study can help to clarify when suspected MODY individuals are misdiagnosed as T1D patients.

In summary, we highlight the significance of *ONECUT1* as a recurrent marker for diabetes. Our data from Arab individuals with diabetes support the involvement of *ONECUT1* variants*,* particularly the rs202151356_p.H33Q, in various forms of diabetes, especially MODY, T1D, and T2D. It is suggested that the same monogenic diabetes gene can contribute to different forms of diabetes with early or late onset, depending on the functional impact of the variant. Likewise, the same pathogenic variant can result in several diabetes phenotypes, even within the same family. It is also possible that patients initially diagnosed with T2D may have monogenic diabetes (Bonnefond et al., 2023). Therefore, there may be a common genetic basis for the development of various forms of diabetes, including T2D. For example, certain T2D susceptibility loci, such as the *PPARG* Pro12Ala variant, *MTNR1B*, *HNF1A*, *GLIS3*, 6q22.32, and novel loci near the MHC, which harbour HLA class II genes, are associated with approximately half of the risk for developing T1D (Eftychi et al., 2004; Raj et al., 2009; Andersen et al., 2014; Mahajan et al., 2014). The *TCF7L2* variants (such as rs7903146 and rs12255372) are associated with the pathophysiology of gestational diabetes mellitus (GDM), T1D, latent autoimmune diabetes of adults (LADA), and T2D (Del Bosque-Plata et al., 2022). The findings of Philippi et al. (2021), Prudente et al. (2022), Russ-Silsby et al. (2023), and our study suggest that *ONECUT1* is potentially associated with clinical diversity in diabetes, such as neonatal syndromic diabetes, insulin-requiring diabetes, insulin-requiring diabetes, MODY, T1D, and early- and late-onset T2D. By way of including the two homozygous mutations (p.Glu231Asp and p.E231X) observed with neonatal diabetes in the works of Philippi et al. (2021), the one homozygous frameshift mutation (p.Met289Argfs*80) observed with neonatal diabetes in the works of Russ-Silsby et al. (2023), and the two homozygous mutations p.H33Q and p.P94P that we observed with MODY, T1D and T2D, a total of 5 homozygous mutations have been reported so far in *ONECUT1* gene as relating to the clinical diversity in diabetes (Supplementary Figure S2).

The present study has certain limitations. Firstly, our study lacked cohorts of patients diagnosed with other types of diabetes such as neonatal diabetes, childhood T2D diabetes, and atypical diabetes. Secondly, the peripheral blood sample is not the ideal biological sample as a) it contains a diverse mixture of cells and DNAm is cell-specific, and b) it may harbour DNA methylation events due to pathological differences relating to autoimmune processes that we could not consider for adjusting the results of differential methylation analysis. However, blood as biological sample has been used in other DNA methylation studies on diabetes (Disanto et al., 2013; Agardh et al., 2015; Johnson et al., 2020; Dashti et al., 2022).

## Conclusion

The data from Arab individuals with diabetes further support the recurrent involvement of *ONECUT1* variants, particularly the homozygous variants of rs202151356_p.H33Q and rs61735385_p.P94P**,** in various forms of diabetes. Additionally, our findings demonstrate the involvement of the *ONECUT1* variants in regulating the transcriptional and epigenetic machinery relating to diabetes.

## Data Availability

The data analyzed in this study is subject to the following licenses/restrictions: The study uses three data sets from previous studies. The first two data sets correspond to minor children diagnosed with MODY and type 1 diabetes—as the participants are minor, their data cannot be made public. However, requests to access these two datasets can be directed to either FA-M (fahd.almulla@dasmaninstitute.org) or TT (alphonse.thangavel@dasmaninstitute.org). The third data set corresponds to adult individuals from Qatar diagnosed with type 2 diabetes; the raw data pertaining to this cohort is available publicly and can be downloaded from the National Center for Biotechnology Information Sequence Read Archive (SRA accessions SRP060765, SRP061943, and SRP061463). Requests to access these datasets should be directed to Requests for the first two data sets: FA-M (fahd.almulla@dasmaninstitute.org) or TT (alphonse.thangavel@dasmaninstitute.org); Requests for the third data set on Qatari T2D individuals: from the National Center for Biotechnology Information Sequence Read Archive (SRA accessions SRP060765, SRP061943, and SRP061463).
