# Supplementary material for: ONECUT1 variants beyond type 1 and type 2 diabetes: exploring clinical diversity and epigenetic associations in Arab cohorts
Source: Front Genet. 2023 Oct 24;14:1254833. doi: 10.3389/fgene.2023.1254833 (PMC10628528; doi:10.3389/fgene.2023.1254833)
Supplement: Supplementary file 1 [file Table1.DOCX]

**Supplementary Table S1.** Transcription factor binding motifs altered by the ONECUT1 variants as inferred using HaploReg tool.

|  | Predicted transcription factor binding motif – Position Weight Matrix | Difference between Reference and Alternate scores (DELTA) | Strand on which the motif is found | Reference score | Alternate score |
| --- | --- | --- | --- | --- | --- |
| rs202151356_p.H33Q |  |  |  |  |  |
|  | Myc_disc9 | -11.34 | **+** | 10.83 | -0.51 |
|  | Myc_known8 | -11.26 | **-** | 12.29 | 1.04 |
|  | YY1_disc4 | -4.12 | **+** | 10.79 | 6.67 |
|  | BHLHE40_disc2 | -3.50 | **+** | 10.66 | 7.16 |
|  | Egr-1_known3 | -2.80 |  | 13.76 | 10.96 |
|  | Egr-1_known2 | -2.41 |  | 11.44 | 9.03 |
|  |  |  |  |  |  |
| rs61735385_p.P94P |  |  |  |  |  |
|  | NF-kappaB_disc2 | -11.97 | **+** | 12.33 | 0.37 |
|  | EBF_known3 | -11.77 | **-** | 5.40 | -6.37 |
|  | Rad21_disc4 | -2.16 | **+** | 13.54 | 11.38 |
|  |  |  |  |  |  |
| 15:53081601:T:G / rs201286990_p.M161L |  |  |  |  |  |
|  | Mef2_known6 | -11.97 | **-** | -10.26 | -22.23 |
|  |  |  |  |  |  |
