# Supplementary figures and images for: ONECUT1 variants beyond type 1 and type 2 diabetes: exploring clinical diversity and epigenetic associations in Arab cohorts
Source: Front Genet. 2023 Oct 24;14:1254833. doi: 10.3389/fgene.2023.1254833 (PMC10628528; doi:10.3389/fgene.2023.1254833)

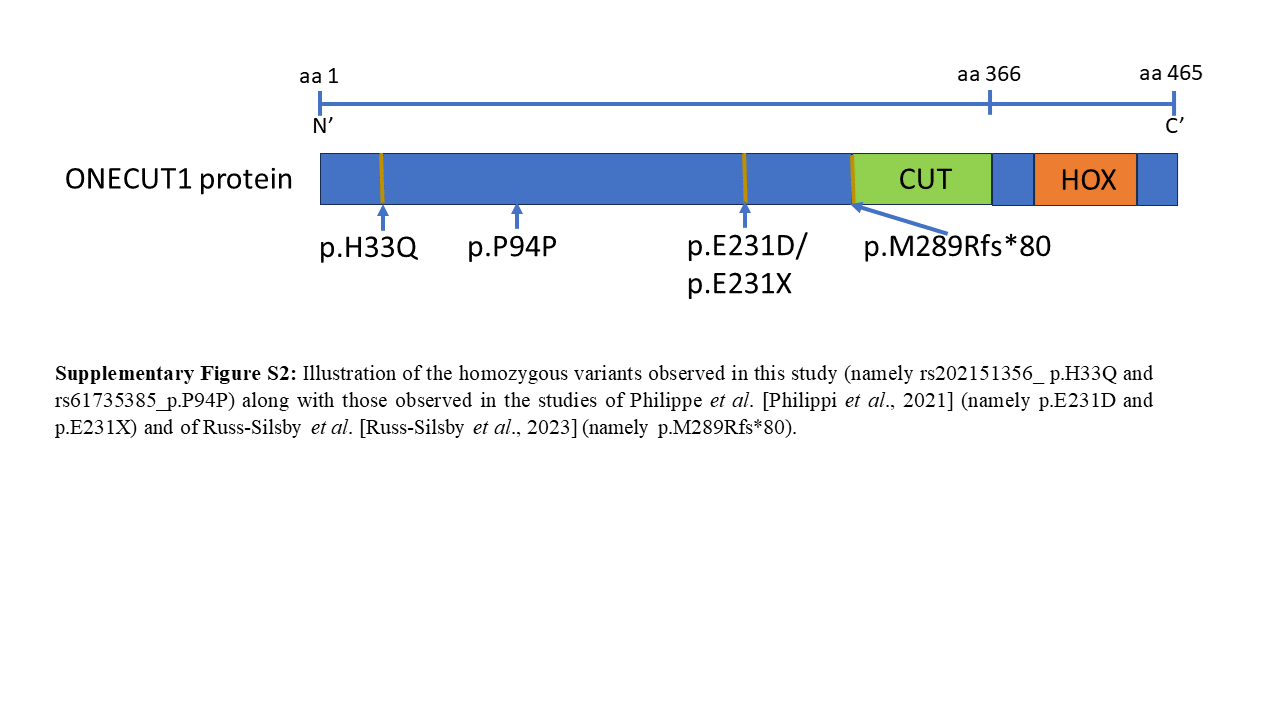

Supplement: Supplementary file 2 [file Image3.TIF]

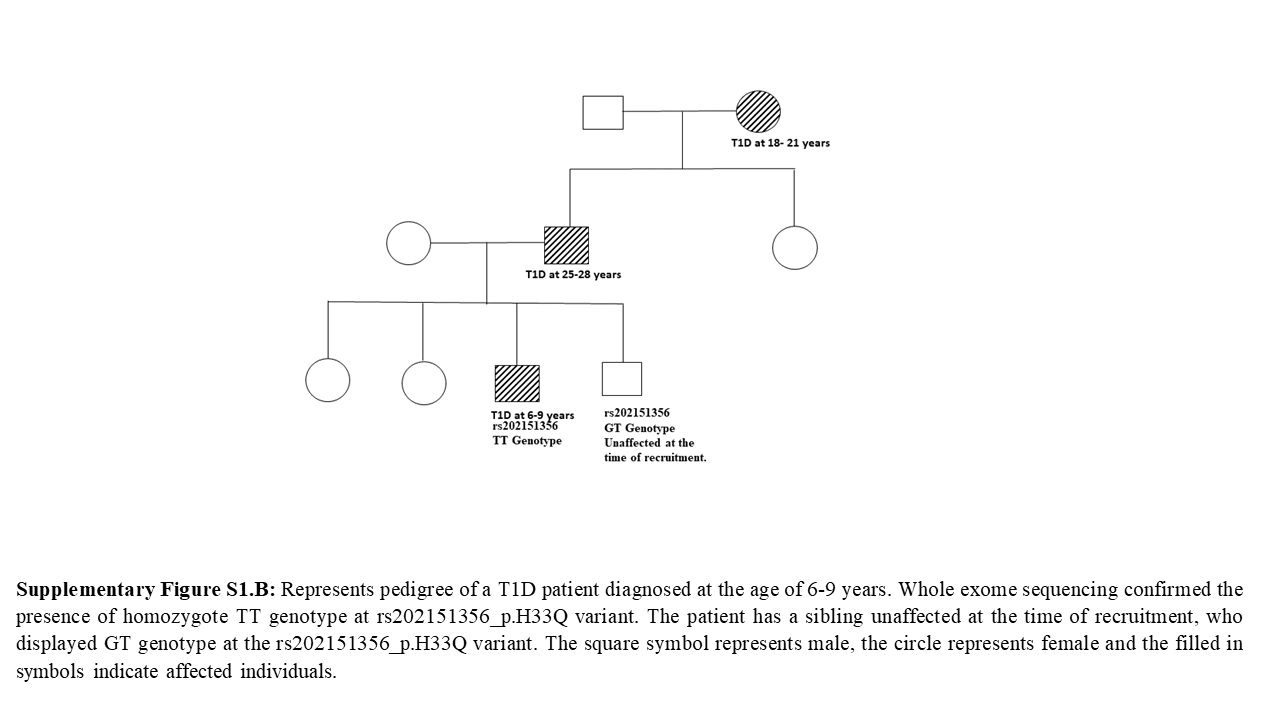

Supplement: Supplementary file 3 [file Image2.TIF]

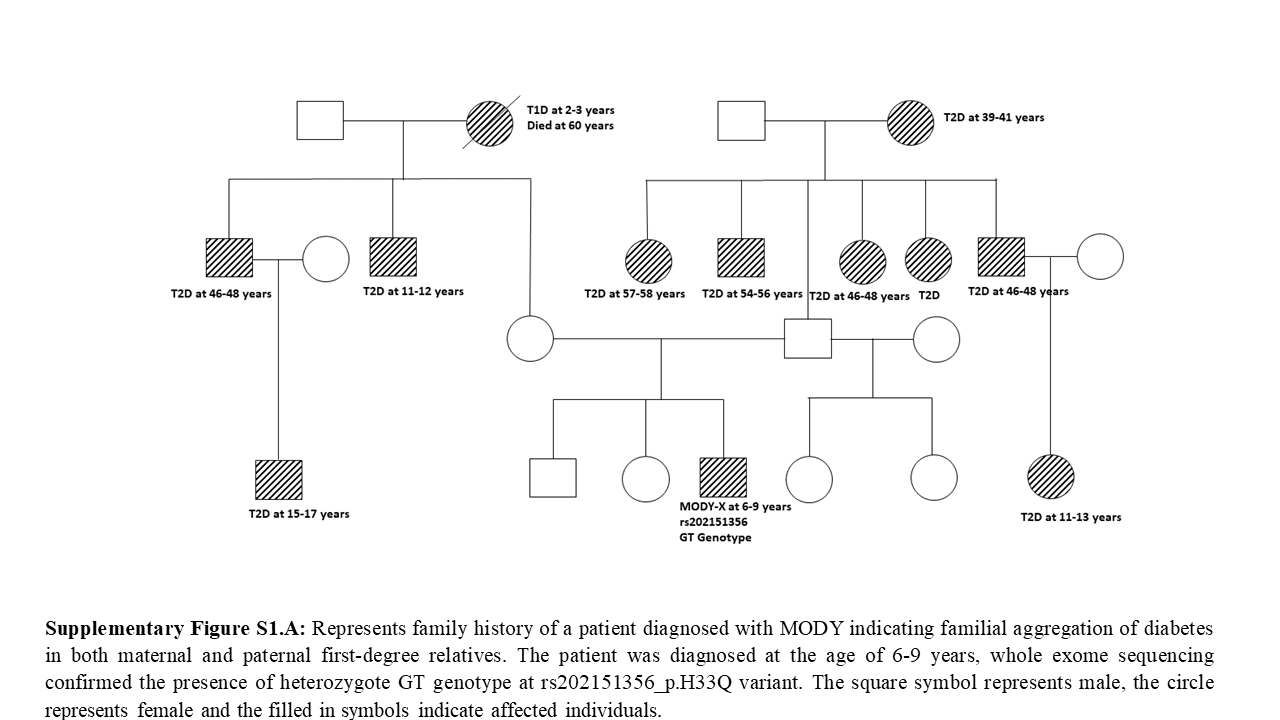

Supplement: Supplementary file 4 [file Image1.TIF]
