# Supplementary material for: ONECUT1 variants beyond type 1 and type 2 diabetes: exploring clinical diversity and epigenetic associations in Arab cohorts
Source: Front Genet. 2023 Oct 24;14:1254833. doi: 10.3389/fgene.2023.1254833 (PMC10628528; doi:10.3389/fgene.2023.1254833)
Supplement: Supplementary file 5 [file Table2.DOCX]

**Supplementary Table S2.** Impact of the observed ONECUT1 variants on differential expression levels of genes.

**(i) As observed from GTEx data**

| Gene | P-value | NES | Tissue |
| --- | --- | --- | --- |
| ONECUT1 | 5.2e-7 | -0.34 | Testis |
| RP11-209K10.2 | 1.1e-6 | -0.50 | Testis |

**(ii) As observed from NephQTL data:**

| Entrez Id | Gene Symbol | dbSNPId | Chr:pos | Ref. | Alt. | Alt. AF | Beta | t-statistic | P-value |
| --- | --- | --- | --- | --- | --- | --- | --- | --- | --- |
| 4644 | MYO5A | rs61735385 | 15:53081800 | G | C | 0.059 | -0.191 | -1.035 | 0.303 |
| 55930 | MYO5C | rs61735385 | 15:53081800 | G | C | 0.0589 | -0.123 | -0.813 | 0.418 |
| 3175 | ONECUT1 | rs61735385 | 15:53081800 | G | C | 0.059 | -0.210 | -0.722 | 0.471 |
| 56204 | FAM214A | rs61735385 | 15:53081800 | G | C | 0.059 | -0.129 | -0.719 | 0.473 |
| 10776 | ARPP19 | rs61735385 | 15:53081800 | G | C | 0.059 | -0.042 | -0.263 | 0.793 |
