# Supplementary material for: ONECUT1 variants beyond type 1 and type 2 diabetes: exploring clinical diversity and epigenetic associations in Arab cohorts
Source: Front Genet. 2023 Oct 24;14:1254833. doi: 10.3389/fgene.2023.1254833 (PMC10628528; doi:10.3389/fgene.2023.1254833)
Supplement: Supplementary file 6 [file Table3.DOCX]

**Supplementary Table S3.** The rs61735385_p P94P variant as QTL for differential methylation of CpG sites in and around ONECUT1 gene as observed in mQTLdb.

| Timepoint | CpG | CpG Pos | beta | t-statistics | Effect Size | p-value | Distance of the CpG site from the position of rs61735385_p P94P variant |
| --- | --- | --- | --- | --- | --- | --- | --- |
| Adolescence | cg02061705 | 15:53082477 | 0.37387 | 5.89742 | 0.00148 | 5.36E-09 | +677 |
| Adolescence | cg11983576 | 15:53090807 | 0.47405 | 7.63214 | 0.00229 | 6.30E-14 | +9007 |
| Adolescence | cg12932613 | 15:53086630 | -0.74426 | -12.1545 | 0.09706 | 2.05E-31 | +4830 |
| Adolescence | cg06824013 | 15:53097247 | 0.33131 | 5.49414 | 0.00231 | 5.22E-08 | +15447 |
| Adolescence | cg06999762 | 15:53093007 | -0.53289 | -8.17327 | 0.07434 | 1.11E-15 | +11207 |
| Adolescence | cg25577821 | 15:53098475 | 0.45336 | 7.25138 | 0.00551 | 9.41E-13 | +16675 |
| Birth | cg06999762 | 15:53093007 | -0.4386 | -6.40693 | 0.04101 | 2.59E-10 | +11207 |
| Birth | cg12932613 | 15:53086630 | -0.67243 | -9.82364 | 0.02942 | 1.55E-21 | +4830 |
| Childhood | cg14492992 | 15:53101548 | 0.33622 | 5.64576 | 0.03004 | 2.26E-08 | +19748 |
| Childhood | cg11983576 | 15:53090807 | 0.51879 | 8.43056 | 0.00143 | 1.51E-16 | +9007 |
| Childhood | cg06999762 | 15:53093007 | -0.59846 | -9.36228 | 0.06254 | 7.10E-20 | +11207 |
| Childhood | cg25577821 | 15:53098475 | 0.48832 | 8.09591 | 0.00341 | 2.01E-15 | +16675 |
| Childhood | cg06824013 | 15:53097247 | 0.36487 | 6.21453 | 0.00396 | 8.13E-10 | +15447 |
| Childhood | cg12932613 | 15:53086630 | -0.74976 | -12.3958 | 0.06349 | 1.68E-32 | +4830 |
| Middle Age | cg11983576 | 15:53090807 | 0.51234 | 7.31176 | 0.0031 | 6.86E-13 | +9007 |
| Middle Age | cg06999762 | 15:53093007 | -0.55173 | -8.15003 | 0.06017 | 1.55E-15 | +11207 |
| Middle Age | cg06824013 | 15:53097247 | 0.43553 | 6.74313 | 0.00431 | 3.12E-11 | +15447 |
| Middle Age | cg12932613 | 15:53086630 | -0.73704 | -11.5114 | 0.09424 | 2.53E-28 | +4830 |
| Middle Age | cg25577821 | 15:53098475 | 0.41441 | 5.84908 | 0.0062 | 7.42E-09 | +16675 |
| Pregnancy | cg14492992 | 15:53101548 | 0.35998 | 5.39653 | 0.01438 | 9.08E-08 | +19748 |
| Pregnancy | cg25577821 | 15:53098475 | 0.47197 | 6.85152 | 0.0055 | 1.51E-11 | -16675 |
| Pregnancy | cg11983576 | 15:53090807 | 0.47294 | 7.4964 | 0.0033 | 1.82E-13 | -9007 |
| Pregnancy | cg06999762 | 15:53093007 | -0.47391 | -6.82894 | 0.05494 | 1.75E-11 | -11207 |
| Pregnancy | cg12932613 | 15:53086630 | -0.74976 | -11.4256 | 0.08797 | 5.13E-28 | -4830 |
| Pregnancy | cg06824013 | 15:53097247 | 0.44568 | 7.07815 | 0.00643 | 3.32E-12 | -15447 |
